# Combination of vascular surgery with novel vascular targeting agents as cancer therapeutics

**DOI:** 10.3389/fonc.2025.1723016

**Published:** 2026-01-23

**Authors:** Jianxin Dong, Ming Sun, Kai Cao, Tao Fang

**Affiliations:** 1 Department of Vascular Surgery, Yantai Mountain Hospital, Yantai, Shandong, China; 2 Vascular Surgery, The Affiliated Taian City Central Hospital of Qingdao University, Taian, China

**Keywords:** anti-angiogenic, oncovascular surgery, pro-angiogenic surge, tumor recurrence and metastasis, vascular targeting agents

## Abstract

Locally advanced solid tumors, characterized by complex involvement or encasement of major vascular structures, present a significant challenge in curative oncology. Achieving microscopically negative margins often mandates extensive surgical procedures, collectively termed Oncovascular Surgery (OVS). While OVS successfully addresses the anatomical barrier to resection, the resulting surgical trauma is intrinsically linked to an acute systemic release of pro-angiogenic factors, frequently correlating with accelerated tumor recurrence and metastatic potential. Novel Vascular Targeting Agents (VTAs) offer critical pharmacological control over the tumor vasculature. These agents are categorized primarily into Anti-Angiogenic Agents (AIAs), which inhibit new vessel growth, and Vascular Disrupting Agents (VDAs), which induce rapid collapse of established tumor blood vessels. The clinical integration of mechanical clearance (OVS) with strategic pharmacological control (VTA administration) is highly complex, demanding precise timing and toxicity management. This review synthesizes the molecular mechanisms underpinning VTA function and selectivity, details the technical necessity and consequences of OVS, and critically evaluates the biological interface including mechanisms of resistance and the systemic post-surgical angiogenic surge to establish a unified translational strategy for synergistic combination regimens.

## 1 Introduction

The vascular compartment of solid tumors functions not merely as a passive supply route but as a dynamic regulatory network that dictates tumor survival, invasiveness, and metastatic potential (1). Modern oncology has recognized the therapeutic potential of targeting this network, moving beyond classical cytotoxic chemotherapy toward molecularly targeted agents. This paradigm shift includes the development of agents designed to control pathological neovascularization (2). For patients afflicted with tumors locally advanced to the extent of major vessel involvement such as soft tissue sarcomas necessitating extremity vessel resection (RSTS/ESTS) or Renal Cell Carcinoma (RCC) with tumor thrombus (TT) extending into the Inferior Vena Cava (IVC) definitive surgical resection is impossible without concomitant vascular intervention (3).

The goal of OVS is unequivocal: to ensure complete tumor clearance, defined by an resection margin. Experts in vascular surgery are essential in planning and executing the major vessel resection and subsequent reconstruction to preserve vital perfusion and drainage (4). However, the efficacy of even the most meticulous surgical resection is often undermined by the unavoidable consequences of surgical trauma, which creates a permissive microenvironment conducive to rapid regrowth of residual disease (5).

The combined use of local control (surgery) and systemic/local vascular modulation (VTAs) is biologically sound because both modalities target symptoms stemming from the same core pathology vascular dysfunction (6). Tumor vasculature is chaotic and poorly regulated, a state that makes it vulnerable to pharmacological disruption by VDAs. This same chaotic state results in tumor hypoxia, which upregulates pro-growth factors like Vascular Endothelial Growth Factor (VEGF), accelerating tumor expansion and often culminating in the vessel infiltration that necessitates OVS. Therefore, success in advanced cancer treatment requires not only the mechanical resolution of vascular encasement via surgery but also the pharmaceutical mitigation of the underlying and post-surgical pro-angiogenic drive (4, 7–9).

## 2 Pathophysiology of the tumor vasculature: a selective target

**Figure 1:**
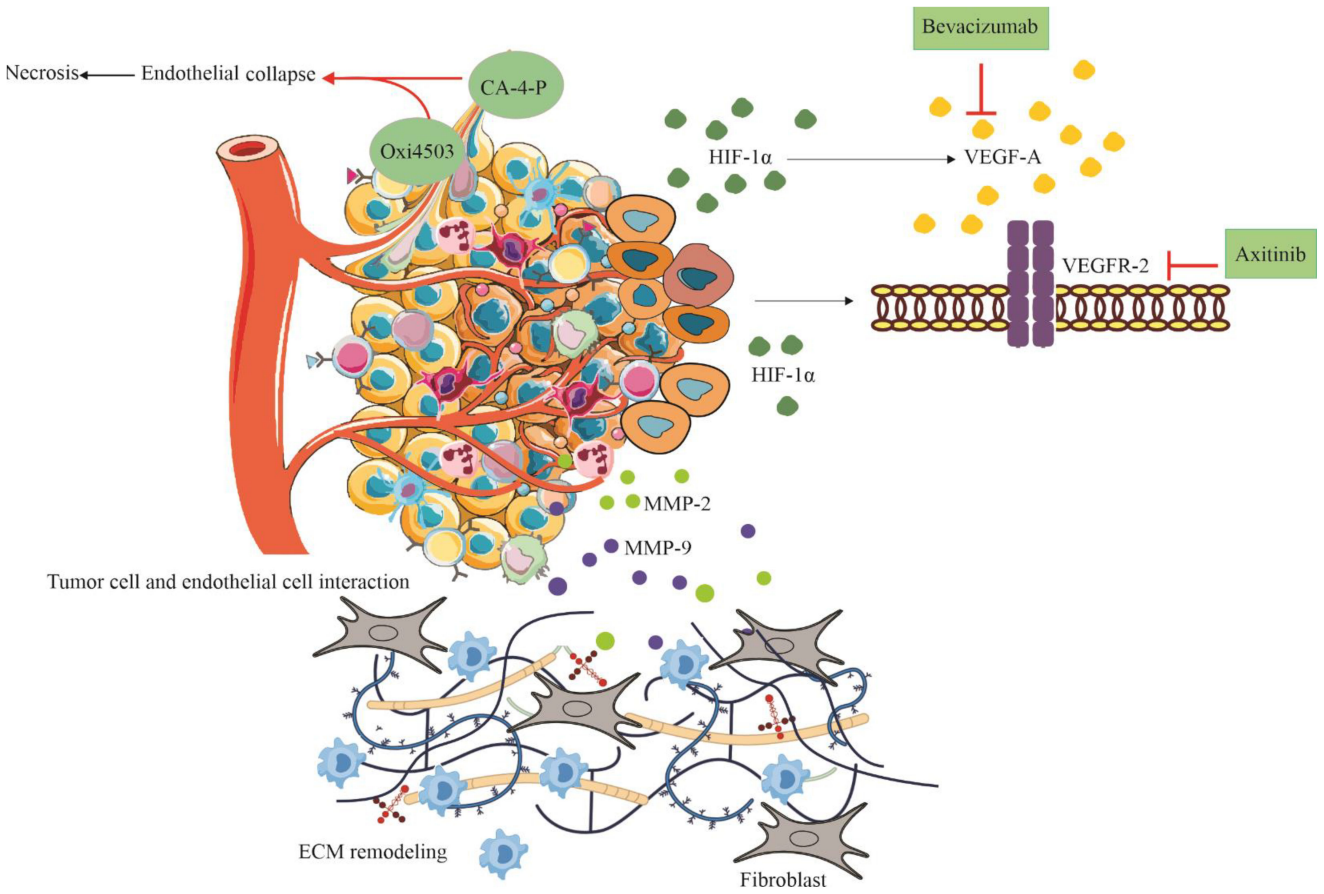
Mechanistic illustration of tumor vasculature and the effects of vascular-targeting agents (VTAs). Hypoxic tumor microenvironment induces stabilization of HIF-1α, leading to VEGF-A secretion and subsequent activation of VEGFR-2 on endothelial cells. VEGFR-2 triggers multiple downstream signaling cascades, including the PI3K/Akt/mTOR pathway, the MAPK/ERK pathway, and Src signaling. Matrix metalloproteinases (MMP-2/9) contribute to extracellular matrix (ECM) remodeling and invasion. Vascular disrupting agents (VDAs) such as CA-4-P and OXi4503 destabilize endothelial microtubules, resulting in endothelial collapse and central tumor necrosis. In contrast, anti-angiogenic agents (AIAs), including Bevacizumab (VEGF-A blockade) and Axitinib (VEGFR-2 inhibition), suppress angiogenic signaling, inhibit neovascular sprouting, and promote vascular normalization.

The vasculature that supplies a malignant tumor exhibits profound structural and functional differences compared to healthy tissues, a characteristic exploited by VTAs (10) (**Figure 1**).

Tumor neo vasculature is typically characterized by a disorganized, aberrant structure featuring excessive vessel tortuosity, irregular caliber, and high permeability (leakiness). Critically, these vessels frequently lack the stabilizing support provided by mature pericyte coverage (11). This immaturity forms the foundational principle of VDA selectivity; the fragility of the endothelial structure in the tumor environment renders it uniquely susceptible to disruption, whereas the more organized and mature endothelial cells in normal host tissues are protected (12).

The physiological consequences of this aberrant architecture include erratic and inefficient blood flow, resulting in zones of chronic and acute tumor hypoxia (13). Furthermore, the vessel leakiness and lack of effective lymphatic drainage contribute to pathologically elevated Interstitial Fluid Pressure (IFP) within the tumor mass. This high IFP acts as a physical barrier, impairing the efficient delivery and uptake of systemically administered chemotherapeutic and cytotoxic agents, thereby driving intrinsic drug resistance (14).

The creation of the abnormal tumor vasculature is overwhelmingly driven by the process of angiogenesis, regulated primarily by the Hypoxia-Inducible Factor-1 (HIF-1) pathway (15). Under hypoxic conditions which are common in fast-growing, poorly perfused solid tumors HIF-1 protein stabilizes and translocates to the nucleus, initiating the transcription of pro-angiogenic genes, notably VEGF-A) (6). VEGF-A is the central, primary angiogenic factor, binding to both VEGFR-1 and VEGFR-2 receptors on endothelial cells, stimulating vessel proliferation and migration (16). This central hypoxia–HIF-1–VEGF axis not only drives pathological angiogenesis but also forms the backbone of resistance, as tumors exploit alternative pathways when VEGF signaling is inhibited.

In addition to VEGF signaling, other molecular pathways are crucial. Matrix Metalloproteinases (MMPs), particularly MMP-2 and MMP-9, play a pivotal role in remodeling the Extracellular Matrix (ECM). By degrading the basement membrane and surrounding stroma, MMPs facilitate endothelial cell sprouting required for angiogenesis. Importantly, MMP activity also releases ECM-bound growth factors, including the proteolytic cleavage and activation of latent Transforming Growth Factor-(TGF-), further fueling tumor progression, metastasis, and angiogenesis (17, 18).

These central angiogenic factors are part of a broader, redundant network. Resistance mechanisms to initial VEGF-A blockade often involve compensatory signaling through alternative receptor systems, including Fibroblast Growth Factors (FGFs), Platelet-Derived Growth Factors (PDGFs), the Angiopoietin/Tie2 axis, and Ephrins. For instance, blockade of VEGF-A/VEGFR signaling often triggers compensatory activation of FGF and PDGF pathways, while Angiopoietin/Tie2 and Ephrin/EphR signaling sustain endothelial survival (19). This redundancy explains why single-agent VEGF inhibition frequently fails and highlights the necessity of multi-target or combination strategies (20). The confluence of chaotic vascular structure, resulting in pervasive hypoxia and the subsequent upregulation of HIF-1 and VEGF, is responsible for generating aggressive tumor behavior that infiltrates adjacent vital structures (21). The vulnerability of these vessels to physical collapse is the target of VTAs. Simultaneously, the rapid growth driven by the hypoxic environment is what necessitates the radical mechanical intervention of OVS. Therefore, OVS and VTA therapy are not distinct treatments but rather complementary interventions addressing different manifestations structural versus systemic of the fundamental pathology of disorganized tumor vascularization (22). In addition to inherent abnormalities in endothelial proliferation, an alternative explanation proposed by Jain and colleagues is that tumor vessels may, in fact, originate as structurally normal vessels but become ‘pulled apart’ as the expanding tumor mass exerts mechanical pressure on the vessel wall. This compressive stress causes pericyte coverage to become stretched thin, functionally mimicking pericyte dropout and contributing to the observed leakiness and immaturity. Incorporating this biomechanical model provides a more complete understanding of why tumor vasculature appears immature and susceptible to VTA action (23).

## 3 Novel vascular targeting agents: classification, mechanism, and scope

Vascular targeting agents encompass two mechanistically distinct pharmacological classes: those that disrupt established vasculature (VDAs) and those that prevent the formation of new vasculature (AIAs). A clear understanding of these mechanisms is paramount for designing rational combination strategies in oncology (11, 24).

VDAs are defined by their ability to induce rapid, widespread, and selective collapse of the existing, abnormal tumor blood supply, leading to acute ischemia, central tumor necrosis, and widespread tumor cell death. This mechanism fundamentally differs from AIAs, which work by starving the tumor over a longer timeframe (25, 26).

The largest and most studied group of small-molecule VDAs are the tubulin-binding agents. These drugs specifically interfere with the microtubule protein system of the endothelial cells (27). The lead compound in this class is Disodium Combretastatin A-4 3-O-Phosphate (CA-4-P or Zybrestat™), which is a water-soluble prodrug. The mechanism of action involves binding to the colchicine site on -tubulin, which inhibits the assembly of tubulin into microtubules (28, 29). This pharmacological effect destabilizes the cytoskeleton of the highly proliferative tumor endothelial cells. The resultant loss of cytoskeletal integrity triggers profound morphological changes in the endothelial lining, including cell rounding, disruption of cell-cell junctions, and ultimately, rapid vascular occlusion and blood flow stasis within minutes to hours of administration (30).

CA-4-P has progressed through Phase I trials, demonstrating systemic tolerability and confirming selective reductions in tumor blood flow consistent with preclinical evidence (31). It has advanced to Phase II/III studies, often tested in combination with radiotherapy, standard chemotherapy (carboplatin, paclitaxel), and AIAs (Bevacizumab) (32). A potent analog, Combretastatin A-1-P (CA-1-P or OXi4503), is considered a more powerful VDA and has shown promising vascular activity in Phase I studies (33).

Despite their effectiveness in inducing massive central necrosis, VDAs typically fail as single agents to halt tumor growth permanently. The primary limitation is that they spare a rim of viable tumor tissue at the periphery, which is perfused by more mature, pericyte-stabilized blood vessels (34). The remaining cells in this viable rim rapidly repopulate the tumor, often stimulated by enhanced angiogenesis triggered by the central necrosis-induced hypoxia (35). This biological failure necessitates combination strategies designed to eliminate or suppress regrowth from the peripheral viable rim using chemotherapy, radiation, or AIAs (36).(**Table 1**) AIAs represent the second major class of VTAs. Their objective is to interfere with the signaling cascades necessary for the sprouting of new blood vessels, a process known as angiogenesis inhibition (37).

**Table 1:** Comparative summary of preclinical and clinical strategies targeting tumor vasculature.

Therapeutic strategy	Cancer type	Agent/class	Treatment protocol	Clinical outcome	Ref.
Pazopanib (VEGFR inhibitor) + Fosbretabulin (vascular disrupting agent)	Ovarian, fallopian tube, and peritoneal carcinomas	Tyrosine kinase inhibitor + tubulin-binding VDA	Surgical resection followed by dual-agent therapy	Improved short-term survival; modest progression-free survival (PFS)	(41)
Combretastatin A4 Phosphate (CA4P) or OXi4503 combined with immune checkpoint inhibitors	Solid tumors (preclinical models)	Vascular disrupting agents + anti-PD-1/PD-L1/CTLA-4	Sequential VDA therapy followed by immune checkpoint blockade	Sensitization of resistant tumors; enhanced immune infiltration and tumor destruction	(42)
Combretastatin A4 Phosphate (CA4P) + standard chemotherapy	Non-small-cell lung cancer (NSCLC)	Tubulin-binding vascular disrupting agent	Surgery combined with CA4P and cytotoxic agents	Enhanced survival within 6–12 months	(43)
Bevacizumab (anti-VEGF monoclonal antibody) + Fosbretabulin	Ovarian and related epithelial tumors	Anti-angiogenic + VDA	Surgery followed by dual vascular-targeted therapy	Significant PFS extension; manageable toxicity	(44)
Ombrabulin (AVE8062) + chemotherapy	NSCLC	Vascular disrupting agent	Surgery plus Ombrabulin and chemotherapy	Mixed survival outcomes; improved local tumor control	(45)
Onco-vascular surgery with preoperative embolization	Musculoskeletal and solid tumors	Vascular occlusion via embolization	Embolization before or during surgery	Reduced intraoperative bleeding; better resection margins	(46)
PEG Hydrogel Embolic System (HES)	Hypervascular solid tumors	Polyethylene glycol-based embolic device	Embolization followed by surgery	High technical success; deep vessel penetration	(47)
Tirapazamine (TPZ) + Hepatic Artery Ligation (HAL)	Hepatocellular carcinoma (HBx transgenic mice)	Hypoxia-activated cytotoxic agent	HAL-induced hypoxia + TPZ (preclinical embolization model)	Selective tumor necrosis; minimal damage to normal liver tissue	(48)
VCAM-1–targeted recombinant fusion proteins (sTF–VCAM-1)	Hodgkin lymphoma, small-cell lung carcinoma (murine and human xenografts)	Coagulation-inducing immunotoxin fusion protein	Systemic administration ± lipopolysaccharide or doxorubicin	Tumor-selective vascular occlusion, necrosis (up to 74%), growth delay, minimal toxicity	(49)
TACE + Percutaneous Microwave Coagulation Therapy (PMCT)	Advanced hepatocellular carcinoma	Locoregional chemoembolization + thermal ablation	Sequential TACE followed by PMCT	Improved survival, reduced recurrence, no added toxicity	(50)
CalliSperes^®^ Drug-Eluting Beads (DEB) + chemoembolization	Solid tumors undergoing TACE	Controlled-release microspheres	Embolization with localized chemotherapy	Dual vessel occlusion; site-specific drug delivery	(51)
CA4P + iRGD-guided nanoparticle therapy	Breast cancer and peritoneal carcinomatosis	VDA + tumor-penetrating peptide-enhanced nanodrug	Sequential vascular disruption and nanoparticle delivery	Enhanced tumor targeting; reduced tumor burden	(52)

The table highlights distinct therapeutic combinations—including VDAs, AIAs, chemotherapy, immunotherapy, and embolization techniques—and their impact on survival outcomes, progression-free survival (PFS), and local tumor control. Notably, dual-agent regimens (e.g., Bevacizumab + Fosbretabulin) consistently demonstrate superior PFS compared to single-agent VDAs, while embolization-based approaches primarily improve surgical feasibility and margin clearance. This comparative overview underscores the importance of multi-target strategies in overcoming resistance and optimizing oncovascular therapy.

The most successful AIAs target the VEGF pathway. Bevacizumab (Avastin) is a foundational monoclonal antibody that blocks the binding of circulating VEGF-A to its receptors (38). Small-molecule Tyrosine Kinase Inhibitors (TKIs), such as Axitinib, target the intracellular components of multiple receptors, including VEGFR-1, VEGFR-2, and VEGFR-3 (39).

A crucial concept associated with AIA therapy, particularly at optimized doses, is vessel normalization. Rather than causing complete vaso-obliteration, controlled inhibition of VEGF signaling can transiently reduce vessel leakiness and hyper-permeability, promoting vessel maturation (40). This temporary normalization improves blood flow, decreases tumor hypoxia, and reduces interstitial pressure, thereby potentially enhancing the delivery and efficacy of subsequently administered cytotoxic drugs or radiation. The vessel normalization window is highly relevant when considering neoadjuvant approaches (23). While VTAs provide pharmacological modulation of tumor vasculature, the mechanical resolution of vessel involvement is addressed in oncovascular surgery, which will be discussed later in the manuscript.

## 4 Principles and technical execution of vascular surgery in oncology

OVS is reserved for locally advanced cancers where the tumor mass directly involves or compresses critical arterial or venous structures, making complete resection impossible without vascular intervention (4). The core surgical indication remains the absolute requirement to achieve an resection, defined as the microscopically complete removal of the tumor mass, as this is the single greatest determinant of survival, outweighing vascular-related complications (53).

Tumor types frequently requiring OVS include retroperitoneal and extremity soft tissue sarcomas (RSTS/ESTS), pancreatic malignancies, and the specialized, high-risk scenario of Renal Cell Carcinoma (RCC) with tumor thrombus (TT) extending into the Inferior Vena Cava (IVC) (54, 55). For ESTS, aggressive vascular resection must be indicated whenever a margin cannot otherwise be achieved. This active approach allows for limb salvage with equivalent oncologic outcomes and significantly improved quality of life (QOL) compared to historic amputation strategies (56, 57). In all complex oncovascular cases, the participation of a vascular surgeon is critical to determine technical resectability, ensure patient safety during tumor extirpation, and facilitate successful revascularization (4).

The technical demands of OVS involve the en-bloc resection of the tumor and the involved vascular segment, followed by immediate, complex reconstruction. Arterial involvement generally necessitates reconstruction through bypassing or the interposition of vascular conduits, which may utilize autogenous vein grafts or various prosthetic materials. While technically demanding, arterial reconstruction is essential to maintain distal organ or limb perfusion (58, 59).

Venous involvement, especially major venous structures like the IVC, poses unique challenges. The approach depends heavily on the level of IVC tumor thrombus (TT) in RCC. For example, resection of right RCC involving an obstructing Level II–IV TT may sometimes be performed without formal reconstruction of the IVC, provided the left renal vein is divided and renal function can be preserved (3, 60). However, in most instances of major vessel invasion, reconstruction or ligation is required. The chosen technique must minimize inadvertent major vessel injury while maximizing the chance of complete tumor removal. Published evidence confirms that overall survival results are dependent upon the achievement of complete clearance and tumor biology, rather than the specific vascular reconstruction method used, validating the principle that major vessel involvement should not preclude curative intent surgery (56, 61).

A vital consideration introduced by the combination approach is the intrinsic tension between the pharmacological benefits of VTAs/AIAs and the necessary safety requirements of major surgery. AIAs, which inhibit the VEGF pathway, are designed to disrupt angiogenesis a process that is also essential for physiological wound healing and endothelial repair (62–64).

The use of neoadjuvant AIAs, such as Bevacizumab, requires meticulous timing and a mandatory washout period (often 4 or more weeks) before surgery to reduce the risks of serious perioperative complications, including anastomotic leaks, impaired wound healing, and hemorrhagic complications (65). This risk analysis demonstrates that surgical feasibility must be continuously balanced against the systemic vascular risks induced by these potent agents. The success of OVS in the neoadjuvant setting relies heavily on the surgical team accurately quantifying and managing the altered vascular biology imposed by the VTA regimen (66).

## 5 The biological interface: timing, trauma, and the angiogenic surge

The rationale for combining OVS with VTAs is strongest when considering the dynamic biological changes induced by surgical trauma in the perioperative period. This interface dictates the optimal timing for VTA administration (neoadjuvant versus adjuvant) (67).

Surgical resection, while curative in intent, is an inherently inflammatory and traumatic event. The trauma, coupled with localized tissue injury and subsequent wound hypoxia at the resection site, triggers a pronounced systemic response. This response involves the rapid and substantial elevation of circulating pro-angiogenic factors, most notably VEGF and Angiopoietin 2 (68, 69).

This phenomenon, commonly referred to as the VEGF surge, is significant, peaking shortly after the procedure and often persisting at elevated levels for up to four weeks (70). The intensity of this angiogenic response correlates with the extent of the surgical wound, suggesting that more extensive OVS procedures might provoke a stronger reaction. The sustained high concentration of these pro-angiogenic proteins creates a highly permissive and nurturing microenvironment, allowing any residual micro-metastases or viable tumor cells to rapidly re-establish a blood supply, thus driving accelerated tumor recurrence (71, 72). This mechanism establishes a definitive, evidence-based argument for utilizing VTA therapy in the adjuvant phase, aimed explicitly at neutralizing this critical window of post-operative vulnerability (73).

Beyond the global angiogenic surge, surgery profoundly alters the local Tumor Microenvironment (TME). The physical disruption of the tumor bed results in transient, severe hypoxia, further stabilizing HIF-1 and initiating a cascade of pro-survival factor upregulation, including VEGF, FGF, and various MMPs (74).(**Table 2**) In addition to VEGF-driven angiogenesis, the FGF and PDGF families play critical and complementary roles in vascular remodeling after oncovascular surgery. FGFs act as potent mitogens that stimulate endothelial cell proliferation, migration, and survival. Importantly, FGF signaling is a well-recognized escape mechanism that becomes upregulated when VEGF pathways are inhibited, allowing tumors to maintain angiogenic capacity despite VEGF blockade (17, 18). Surgical trauma and wound hypoxia further amplify FGF release from fibroblasts and stromal cells, enhancing neovascular sprouting during the early post-operative period.

**Table 2:** The biological interface impact of tumor microenvironment on VTA/AIA timing.

Biological event/marker	Timing relative to surgery	Physiological mechanism	Consequence for tumor recurrence	Optimal VTA/AIA timing strategy	Ref
HIF-1α/VEGF pathway activation under hypoxia	Post-radiotherapy or post-hypoxia	Hypoxia upregulates HIF-1α, which induces VEGF-A expression and promotes angiogenesis in residual tumor tissue	Enhances neovascularization, supports survival of residual tumor cells, and may reduce radiotherapy efficacy	Post-radiotherapy AIA to suppress VEGF surge; neoadjuvant anti-HIF-1α to precondition tumor vasculature	(85)
Tumor vessel maturation and pericyte coverage	Preclinical vascular development	Tumor vessels develop in stages; immature sprouts lack pericytes and are highly proliferative; mature vessels are stable	Immature, pericyte-negative vessels are more susceptible to anti-vascular therapy; mature vessels resist	Target immature vasculature with VTAs during early tumor growth; assess pericyte coverage to guide timing	(86)
VDA-induced receptor upregulation enables nanotherapy	Post-VDA molecular sensitization phase	VDA (CA4P) upregulates αv-integrins and NRP-1 in peripheral tumor tissue, enhancing nanoparticle penetration via iRGD	Enables targeted delivery of Utorubicin-loaded polymersomes; reduces tumor burden without added toxicity	Sequential targeting: VDA to induce receptor expression, followed by iRGD-potentiated nanotherapy	(52)

PDGF signaling contributes to the stabilization and maturation of newly formed vessels by recruiting and activating pericytes. After surgical debulking, PDGF-B/PDGFR-β signaling increases in the regenerating wound margin, promoting both vascular repair and the survival of residual tumor vessels. This process may inadvertently protect pericyte-rich peripheral vessels (31). The integration of FGF- and PDGF-mediated compensatory pathways underscores why mono-targeted VEGF inhibition may be insufficient in the post-operative angiogenic surge and supports the rationale for combination or multi-target approaches.

Furthermore, significant tumor debulking, particularly in high-volume diseases like advanced epithelial ovarian cancer, is associated with a detrimental shift in the local immune landscape. Studies indicate that debulking surgery tends to increase immunosuppression, characterized by the upregulation of immune inhibitory factors such as Interleukin-10 (IL-10) and the downregulation of immunostimulatory factors (75, 76). This surgically induced immunosuppressive environment can impede endogenous immune clearance and may contribute to the failure of VDAs or AIAs, whose mechanisms often rely on an effective immune response or at least a neutral TME (77, 78).

**Figure 2:**
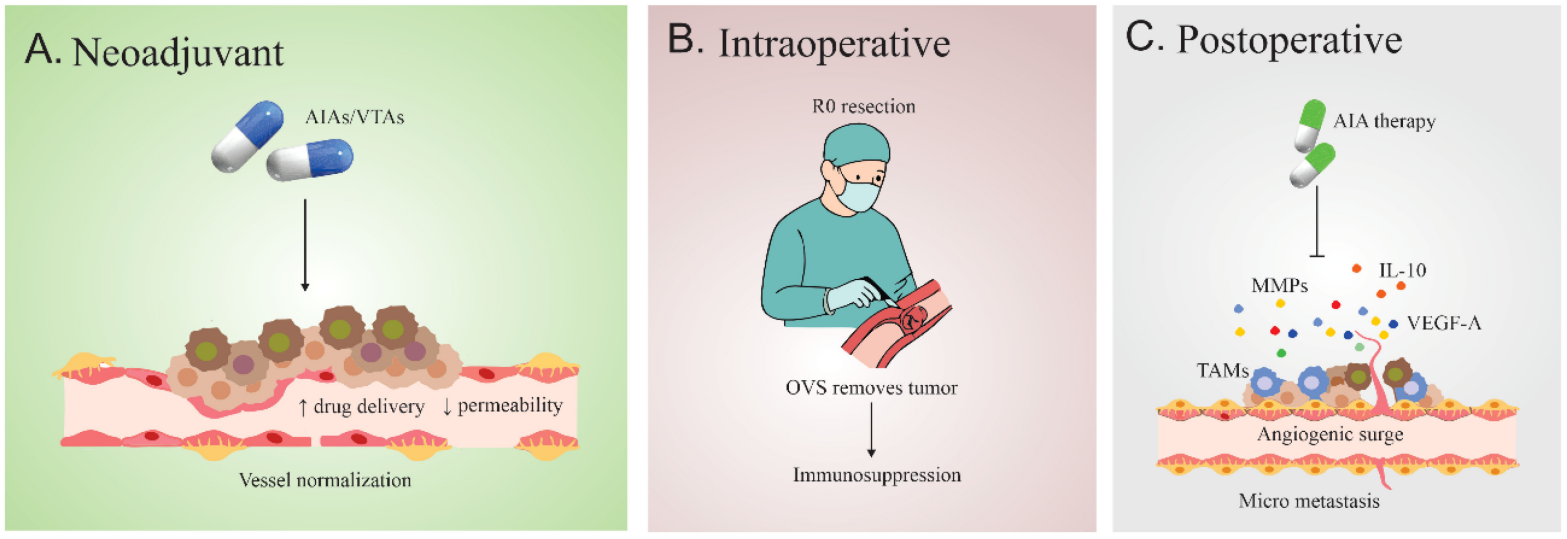
Trimodality treatment concept **(A)** Neoadjuvant AIAs/VTAs normalize tumor vasculature, reducing permeability and enhancing drug delivery. **(B)** Intraoperative OVS achieves R0 resection but induces an angiogenic–immunosuppressive surge. **(C)** Postoperatively, VEGF-driven recruitment of CEPs and TAM-mediated secretion of VEGF-A, MMPs, and IL-10 promote micrometastasis; adjuvant AIA therapy counteracts this response.

In the neoadjuvant setting, VTA/AIA therapy is administered pre-operatively. The primary goal is cytoreduction shrinking the bulk tumor or, specifically in RCC, reducing the tumor thrombus burden within the IVC to simplify the surgical procedure and enhance the likelihood of an resection (79). AIAs are well-suited here, as they may achieve vessel normalization, potentially improving the delivery of concurrent neoadjuvant chemotherapy or radiation, thereby shrinking the tumor mass (10), (**Figure 2**).

The adjuvant model focuses on administering VTAs/AIAs immediately post-operatively (80). Given the compelling evidence for a sustained, systemic pro-angiogenic surge following resection, the adjuvant approach aims to suppress this biological signal, denying residual microscopic disease the necessary vascular supply for rapid regrowth. However, this strategy is complicated by the risk of compromising wound healing due to VTA/AIA interference with essential vascular repair pathways (81, 82).

The intrinsic complementarity between VDAs and AIAs suggests a maximally effective trimodality sequence: a VDA pulse (targeting the established, vulnerable core vasculature) followed by OVS (removing the necrotic core and peripheral rim), and then maintenance AIA therapy (suppressing the post-surgical angiogenic surge) (83, 84). VDAs achieve massive central kill but leave a viable rim. Immediate OVS eliminates this surviving rim, providing maximal local clearance (11, 52).

Surgical resection induces a profound reshaping of the tumor immune TIME, characterized by rapid release of cytokines that serve dual roles in initiating wound repair while simultaneously creating conditions permissive to tumor cell survival. A key distinction must be made between the pre-operative TIME and the post-operative TIME, in which acute inflammation and wound-healing responses dominate.

Interleukin-6 (IL-6) rises sharply within hours of surgery as part of the systemic inflammatory response syndrome (SIRS) (66). Beyond its role in hepatic acute-phase signaling, IL-6 promotes STAT3 activation in residual tumor cells, enhancing proliferation, angiogenesis, and epithelial–mesenchymal transition. Tumor necrosis factor-α (TNF-α) is similarly elevated, contributing to early wound healing but also driving MMP activation, extracellular matrix remodeling, and endothelial activation.

Interleukin-10 (IL-10), although traditionally considered immunosuppressive, plays a necessary regulatory role in suppressing excessive inflammation that would otherwise impair wound healing. Its elevation following debulking surgery does not merely reflect immune suppression but represents a compensatory mechanism to prevent uncontrolled tissue damage (73). However, persistently high IL-10 levels have been associated with increased tumor cell migration and reduced anti-tumor immunity (74).

Transforming growth factor-β (TGF-β) is another critical mediator that rises in the post-operative milieu, promoting fibroblast activation, collagen deposition, and neovascular maturation while simultaneously suppressing cytotoxic T-cell activity. These cytokine shifts interact with VEGF- and Ang-2–driven angiogenesis (68, 72), creating a unique post-surgical window in which anti-angiogenic agents may counteract the rebound vascularization but must be timed carefully to avoid impairing physiological wound repair.

Taken together, the biological interface outlined above emphasizes that timing of VTA administration is not only a theoretical consideration but a practical determinant of clinical success. Translating these molecular and immunological insights into perioperative practice requires clear scheduling guidelines. In the preoperative (neoadjuvant) setting, anti-angiogenic agents such as Bevacizumab may be used to normalize tumor vasculature and reduce thrombus burden, but a washout period of at least six weeks prior to surgery is essential to minimize risks of impaired wound healing and bleeding (87). During the intraoperative phase, vascular disrupting agents can be applied as short-pulse therapy to reduce tumor perfusion and facilitate resection, provided that ischemic injury to surrounding normal tissues is carefully monitored. In the postoperative (adjuvant) period, anti-angiogenic therapy may be reintroduced two to four weeks after surgery, once adequate wound healing is confirmed, to counteract the VEGF surge and prevent rapid revascularization of residual tumor cells. These practical intervals integrate the biological rationale with surgical safety, offering clinicians a framework to optimize VTA scheduling across the perioperative continuum. Beyond surgical integration, VTAs can also be incorporated into multimodal frameworks with chemotherapy and immunotherapy. Anti-angiogenic agents administered during the vessel normalization window may enhance cytotoxic drug delivery, while vascular disrupting agents can increase tumor antigen release, thereby potentiating immune checkpoint blockade. Such synergistic scheduling underscores the potential of VTAs not only as surgical adjuncts but as central components of comprehensive oncovascular therapy.

## 6 Preclinical and mechanistic evidence for combined strategies

Preclinical research strongly supports the concept of dual vascular targeting, recognizing that VDAs and AIAs address distinct spatial and temporal challenges within the tumor mass. VDAs operate acutely, collapsing the established vessels in the tumor core. AIAs, in contrast, operate chronically, preventing the formation of new vessels at the tumor periphery (11).

Studies combining tubulin-binding VDAs, such as CA4P or OXi4503, with the anti-VEGF antibody Bevacizumab (an AIA) demonstrated enhanced anti-tumor activity in human clear cell renal carcinoma xenografts (88).

Preclinical models assessing the combination of VDAs/AIAs primarily focus on integrating them with chemotherapy or radiation therapy, targeting the viable peripheral tumor rim often left after initial VDA therapy. These studies emphasize the critical importance of treating the residual disease to prevent rapid repopulation (89).

However, detailed preclinical evidence explicitly linking VDAs with mechanical *surgical* debulking to assess R0 rates, wound healing, or the suppression of the post-operative angiogenic surge remains an area of translational deficit within the published literature.^10^ The strong evidence for VDA synergy with chemotherapy and radiotherapy suggests, by extension, a potential benefit when combined with surgery, provided that the surgical intervention effectively removes the residual tumor rim, thereby completing the VDA’s cytoreductive effect (11).

The systemic physiological changes induced by surgery the high circulating levels of VEGF and Ang-2 function biochemically as a potent mechanism of VTA resistance. Elevated VEGF drives the recruitment of bone marrow-derived circulating endothelial progenitor cells (CEPs) (90–92). Preclinical studies have reported a transient surge in circulating bone marrow-derived progenitor cells following administration of vascular disrupting agents such as CA-4-P, a response likely driven by compensatory upregulation of VEGF. While some models particularly in neonatal settings demonstrate VEGF-facilitated engraftment of endothelial progenitors into neovasculature, adult systems appear resistant to such incorporation. This dichotomy underscores a critical therapeutic insight: surgical intervention may rapidly reshape the tumor microenvironment into a pro-recruitment niche, rich in angiogenic signals and stromal mobilization cues. Consequently, the adjuvant VTA phase must be strategically intensified to neutralize this multifactorial rebound, targeting both vascular regeneration and cellular infiltration pathways potentiated by the operation itself (93, 94).

## 7 Clinical integration and translational outcomes

The most significant clinical examples of integrating vascular targeting agents with complex surgery involve tumors that invade major vessels, particularly RCC with IVC TT. Up to 25% of RCC patients present with tumor thrombus extension into the IVC, correlating with a poor prognosis if untreated. Complete surgical removal of the tumor and thrombus often requires highly complex OVS with IVC exposure and reconstruction, especially for Level III and IV thrombi (3).

Current clinical trials are actively testing neoadjuvant targeted therapy to improve surgical outcomes. For instance, a Phase II trial is evaluating the use of the TKI Axitinib (AIA) combined with the immune checkpoint inhibitor Pembrolizumab in patients with RCC and associated IVC TT. The goal of this neoadjuvant approach is to utilize the anti-angiogenic and immunotherapeutic effects to decrease the size and invasiveness of the tumor thrombus, potentially reducing surgical complexity, minimizing complications, and improving long-term outcomes (95).

Dedicated Phase II studies, such as NCT00113217, have investigated neoadjuvant Bevacizumab (AIA) therapy in RCC, aiming to assess efficacy, toxicity, and impact on pathological response. The protocol involves a defined initial phase of Bevacizumab administration followed by surgery, and then adjuvant continuation based on response and tolerability. Crucially, the protocol stipulates that surgery must occur at least 4 weeks after the final Bevacizumab dose. This strict washout period underscores the perceived risk associated with AIA activity during the critical window of perioperative wound healing (96). Monitoring during these neoadjuvant regimens involves frequent assessments of serum markers (VEGF, MMP-2, MMP-9) and rigorous safety checks, especially for hypertension and proteinuria (96–98). The combined modality approach is intrinsically constrained by the overlapping toxicity profiles of the targeted agents and the risks associated with major OVS.As noted, anti-VEGF agents interfere with the fundamental biological process required for vascular repair and wound healing. Early postoperative anti-angiogenic therapy, while theoretically beneficial for neutralizing the angiogenic surge, carries significant safety concerns regarding graft patency, anastomotic integrity, and overall recovery. Further research is required to define the long-term effects and safety profile of restarting these therapies in the immediate post-operative period (99, 100), (**Table 3**).

**Table 3:** Summary of key clinical trial archetypes integrating VTA/AIA therapy and definitive oncovascular surgery.

Study focus (archetype)	Cancer type/surgical indication	Vascular agent/modality	Treatment schedule	Primary endpoint(s) addressed	Key clinical implication	Ref
Neoadjuvant systemic downstaging of venous tumor thrombus	Renal cell carcinoma (RCC) with IVC tumor thrombus to facilitate radical nephrectomy ± thrombectomy	Axitinib (VEGFR TKI) + Pembrolizumab (PD-1 inhibitor) systemic TKI + IO combination	Preoperative induction therapy (defined cycles of axitinib + pembrolizumab prior to surgery)	- Radiographic thrombus regression/level reduction - Operative resectability and perioperative safety (R0 resection rate, complications)	Strategy to reduce surgical complexity and high-level thrombus burden.	(102)
Real-world feasibility and safety study	RCC with tumor thrombus (TT)	Mixed neoadjuvant therapies (unspecified agents)	Neoadjuvant prior to radical nephrectomy + thrombectomy	Perioperative parameters, postoperative complications, OS, CSS	Safe and feasible; no significant OS/CSS difference; higher transfusion rate; comparable complication rateperiods to manage surgical risk.	(103)
VDA Combination (ASA404 Trials)	Advanced Solid Tumors (e.g., NSCLC)	ASA404 (VDA) + Chemotherapy	Concurrent (Non-surgical)	Overall Survival, Response Rate	Validated the principle of combining VDAs with standard systemic therapy to overcome VDA resistance.	(104)

VDAs, particularly the microtubule-depolymerizing agents like CA-4-P, are associated with acute cardiovascular toxicity, including myocardial ischemia and reversible neurological events.^5^ These risks are manageable in the general cancer population but represent a substantial complication for patients undergoing major, high-risk OVS, especially those with existing cardiovascular comorbidities typical of advanced cancer patients. This cardiovascular risk necessitates careful patient selection and specialized monitoring when integrating VDAs into the perioperative setting (27, 101).

## 8 Mechanisms of VTA resistance and strategies to overcome them

The development of resistance, whether intrinsic or acquired, severely limits the durability of vascular targeting strategies. Understanding how tumors evade vascular control is essential for sustaining the benefits achieved by combining therapy with surgery.

The primary route of acquired resistance to AIAs, particularly those targeting VEGF-A, is the compensatory upregulation of alternative or redundant pro-angiogenic factors. These factors, including Fibroblast Growth Factors, Angiopoietin-1, and Ephrins, can sustain the endothelial proliferation and survival necessary for tumor perfusion despite effective VEGF blockade (105, 106). This complexity mandates shifting therapeutic focus toward multi-kinase inhibitors capable of targeting broader signaling networks or combination regimens that neutralize multiple pathways concurrently (107).VDAs and AIAs themselves can induce resistance mechanisms. By causing acute ischemia, VDAs induce severe hypoxia in the surviving rim, which acts as a powerful trigger for HIF-1 stabilization and the release of pro-survival factors. This hypoxia-driven TME shift contributes directly to resistance (108).

Furthermore, the tumor response involves the active recruitment of pro-survival cell types. Bone marrow-derived circulating endothelial progenitor cells (CEPs) and Tumor-Associated Macrophages (TAMs) are recruited to the ischemic tumor site, driving VEGF-independent angiogenesis that restores tumor blood supply. The observed acute rise in circulating bone marrow progenitors post-VDA pulse reinforces the need to target these compensatory cellular recruitment mechanisms (109).

The inherent limitations of single-agent VDAs/AIAs necessitate combined strategies, particularly when integrated with OVS. The highly synergistic preclinical results of combining VDAs and AIAs (dual vascular targeting) provide a robust platform for overcoming resistance by covering both the established core and the proliferating periphery (110, 111).

Moreover, both surgical debulking and VTA therapy can influence the tumor immune microenvironment. Surgery may push the TME toward an immunosuppressive state, while hypoxia induced by vascular targeting may also hinder effective immune cell function. Therefore, combining VTAs/AIAs with modern immunotherapies (e.g., PD-1/PD-L1 blockade) represents a promising strategy to counteract the immunosuppressive TME induced by both the local vascular intervention and the systemic surgical trauma (112, 113).

The systemic pro-angiogenic surge resulting from OVS constitutes a powerful, transient, extrinsic resistance mechanism that accelerates recurrence by rapidly revascularizing residual disease. This surge, driven by elevated VEGF, Ang-2, and MMPs, mirrors the biochemical pathways utilized by tumors escaping VTA therapy. Consequently, immediate adjuvant VTA/AIA therapy is not just an anti-cancer measure, but an essential anti-resistance strategy required to neutralize the adverse biological legacy of the surgical procedure itself (114, 115).

The use of anti-angiogenic medicines in the immediate post-operative phase, ahead of conventional chemotherapy or radiation, has been theoretically proposed to suppress this pro-angiogenesis response (116, 117). However, careful monitoring for wound and anastomotic healing complications must accompany such aggressive adjuvant schedules.

## 9 Future directions and emerging translational opportunities

The path forward for combining OVS and VTAs requires overcoming challenges related to systemic toxicity, resistance, and the optimal timing of administration. The mandatory suspension of systemic VTAs/AIAs during the critical perioperative period severely limits their potential to prevent recurrence in the immediate post-surgical window. LDDS offers a groundbreaking solution by allowing for high concentrations of VTAs to be delivered site-specifically to the tumor bed or along reconstructed vascular conduits while minimizing systemic exposure and associated toxicity (24, 118).

Nanoparticle-based formulations, biodegradable polymer hydrogels, or implanted drug-eluting devices could be applied directly to the margins of the resection cavity following OVS. This localized delivery could effectively suppress the *local* VEGF surge and prevent early local recurrence without compromising systemic wound and anastomotic healing. Research utilizing phage display has already identified specific molecules expressed on the surface of tumor endothelial and perivascular cells, providing molecular targets ready for integration into ligand-directed LDDS (119, 120).

To circumvent the problem of redundant signaling pathways a hallmark of acquired AIA resistance future research must expand therapeutic targets beyond the classical VEGF/VEGFR system. Potential novel targets include regulators of vessel maturation and stability, such as the Angiopoietin/Tie2 axis, or specific components of the Notch signaling pathway (121).

Metabolic targeting is also emerging as a viable strategy. Recent studies have highlighted the importance of endothelial glycolytic pathways in neovascular diseases. Targeting specific metabolic dependencies within the proliferating tumor endothelium could provide novel pharmacological agents that are highly selective and complementary to microtubule disruption or growth factor inhibition (122, 123).

The success of a combined regimen depends on selecting patients who are most likely to respond to VTA therapy. This requires the identification of predictive biomarkers. Analysis of gene expression profiles, whether from the primary tumor or the IVC tumor thrombus itself, could predict sensitivity or inherent resistance to specific AIAs or VDAs (124, 125). Furthermore, resistance monitoring must become real-time. Advanced imaging technologies, such as specialized Magnetic Resonance Imaging (MRI) markers, are essential for monitoring VDA-induced vascular collapse and for detecting signs of early resistance, such as increased microvascular density or compensatory perfusion pathways. Integrating these imaging biomarkers into the perioperative workflow will allow clinicians to adjust VTA timing or dosage dynamically, thereby guiding subsequent surgical decisions or pharmacological strategies (126). Emerging biological mechanisms may further strengthen vascular-targeted drug combinations. Protein post-translational modifications (PTMs) regulate immune checkpoints and tumor immunity (127). Caspases and cell death receptors show multifunctional roles beyond apoptosis, offering new therapeutic angles (128). ATP-induced cell death provides prognostic value and potential metabolic vulnerabilities in cancer (129). Non-coding RNAs (miRNAs, lncRNAs, circRNAs) act as master regulators of angiogenesis and resistance, opening opportunities for integration into multimodal oncovascular frameworks (130). Together, these technological innovations and emerging biological mechanisms highlight a multidimensional roadmap for vascular-targeted drug combinations, integrating delivery systems, metabolic interventions, and novel molecular regulators to achieve durable and comprehensive cancer control.

## 10 Conclusion

The combination of advanced vascular surgery and novel vascular targeting agents represents the next critical step in curative therapy for locally advanced, highly vascular malignancies. Meticulous Oncovascular Surgery ensures local clearance, which is paramount for survival. However, this mechanical intervention necessitates a sophisticated biological counterbalance to mitigate the unavoidable systemic and local pro-angiogenic surge that otherwise promotes rapid tumor recurrence.

The strategic timing of VTA administration is the most salient translational challenge. Neoadjuvant therapy (e.g., Bevacizumab or Axitinib) offers the opportunity for vessel normalization and tumor cytoreduction, potentially converting unresectable lesions into operable ones and simplifying complex OVS. Conversely, adjuvant therapy is mandated by the need to suppress the surgically induced pro-angiogenic environment, acting as an essential anti-resistance mechanism.

Future translational success hinges on four key areas: 1. the strategic integration of VDAs (for acute core collapse) with AIAs (for chronic peripheral suppression), maximizing coverage against the heterogeneous tumor vasculature; 2.the development and clinical adoption of localized drug delivery systems to bypass systemic toxicity and maximize drug concentration at the high-risk resection margins. 3) dedicated, prospective clinical trials that rigorously define the optimal timing, dose, and safety profile of VTAs in the immediate perioperative window; and 4. the discovery and validation of predictive biomarkers to personalize agent selection and optimize therapeutic efficacy against acquired resistance mechanisms. Only through this synergistic integration of anatomical expertise and biological control can maximal curative potential be realized for patients facing complex oncovascular disease.
